# Supplementary material for: Semaglutide ameliorates cardiac remodeling in male mice by optimizing energy substrate utilization through the Creb5/NR4a1 axis
Source: Nat Commun. 2024 Jun 4;15:4757. doi: 10.1038/s41467-024-48970-2 (PMC11150406; doi:10.1038/s41467-024-48970-2)

## SUPPLEMENTAL MATERIAL

### Supplemental Figures

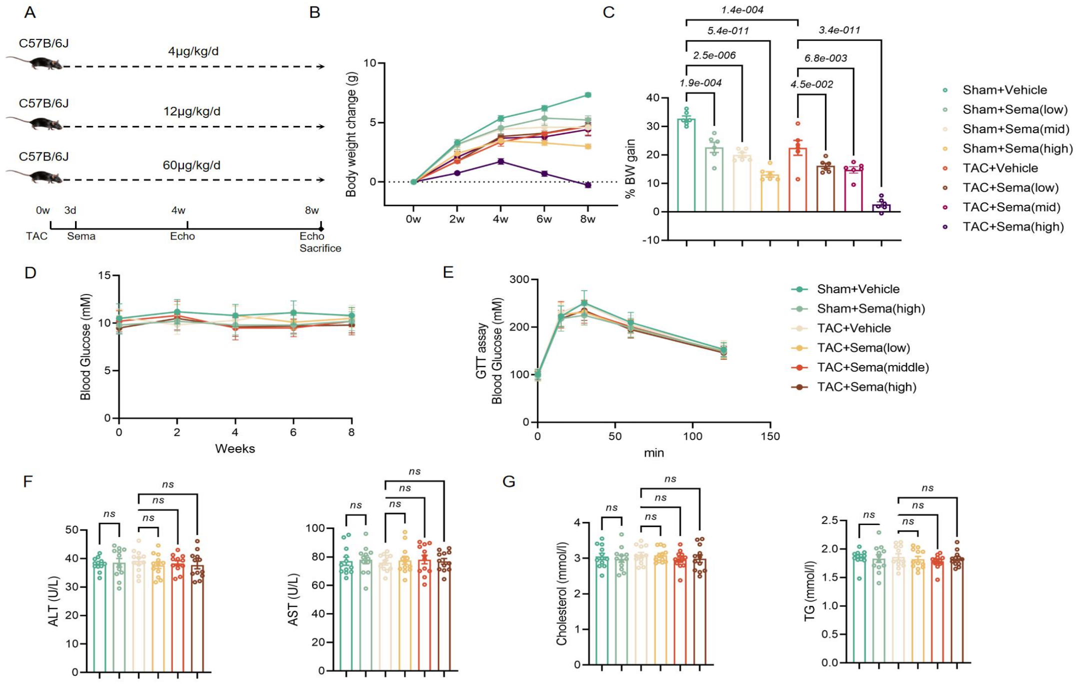


**Figure S1. Effects of different doses of Sema on body weight and glycolipids in mice that underwent TAC for eight weeks.** (A) Mouse model intervention timeline diagram. (B-C) Effect of different doses of Sema on body weight of mice after sham or TAC surgery n=6; BW gain: F (5, 66) = 4.917, P = 0.0007. (D) Effects on fasting blood glucose in mice treated with different doses of Sema every two weeks after sham or TAC surgery (n=6). (E) The results of the GTT after eight weeks of treatment with different doses of Sema n=6. (F) Detection of the liver function indicators ALT and AST after eight weeks of treatment with different doses of Sema (n=12); ALT: F (5, 66) = 0.2430, P = 0.9419; AST: F (5, 66) = 0.09683, P = 0.9924. (G) Measurement of total cholesterol and triglyceride levels after eight weeks of treatment with different doses of Sema (n=12); Cholesterol: F (5, 66) = 0.4508, P = 0.8112; TG: F (5, 66) = 0.3203, P = 0.8991. All results are shown as the mean ± SEM, and analysis using one-way ANOVA followed by Bonferroni post hoc test (C and F-G) was conducted. For the analysis in (B and D-E), repeated measures two-way ANOVA followed by Sidak post hoc test was conducted. p values are indicated. Source data are provided as a Source Data file.TAC, transverse aortic constriction; GTT, glucose tolerance test; TG, triglycerides; ALT, liver enzymes alanine aminotransferase ;AST, aspartate aminotransferase.

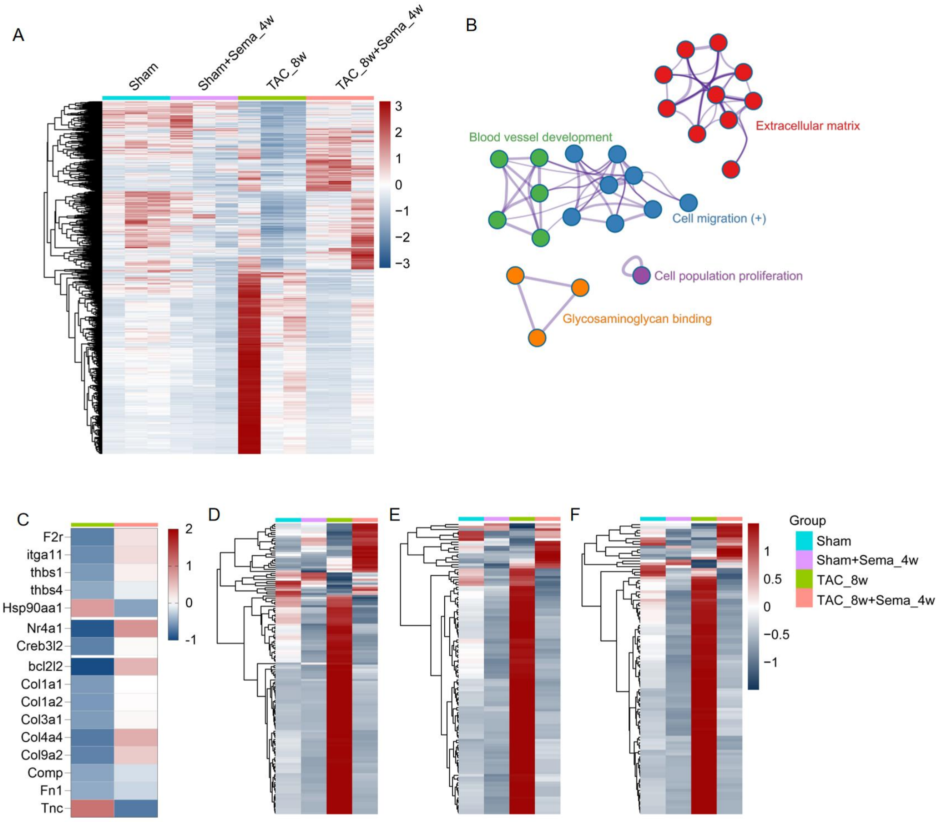


**Figure S2. Sema reverses cardiac hypertrophy, fibrosis and dysfunction in mice induced by TAC by regulating ECM deposition, cell migration, and vascular development pathways.** (A). Heat map of gene level changes in the sema reversal experiment, n=3. (B). Gene Set Enrichment Analysis (GSEA) analysis network maps show key molecular pathways involved in the cardiac remodeling process and regulated by Sema n=3. (C). Heat map showing key molecular signatures both involved in the cardiac remodeling process and regulated by Sema, n=3. (D-F). Heatmap showing changes in genes involved in the ECM deposition, cell migration, and vascularization pathways involved and regulated by Sema during the reversal experiments, n=3. All results are shown as the mean±SEM. p values are indicated.

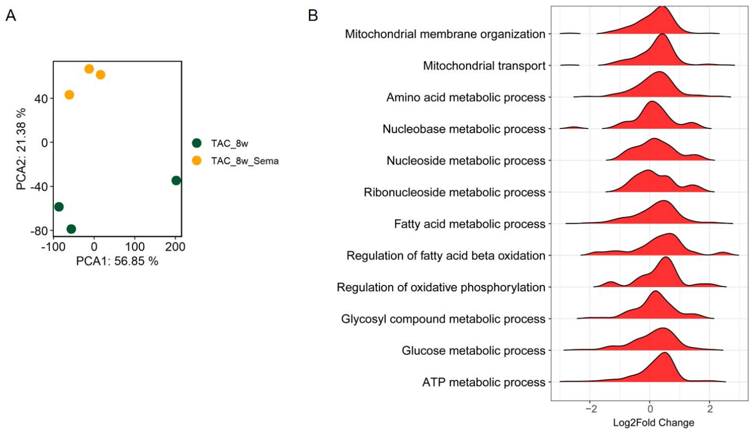

**Figure S3. Transcriptome analysis of Sema ameliorates energy metabolism in pathological cardiac remodeling.** (A) Cardiac tissues were harvested for transcriptome analysis. PCA (principal component analysis) is shown (n=3). (B) Gene set enrichment analysis (GSEA) of mitochondrial function and energy metabolism-related pathways identified 12 significantly altered pathways (n=3). All results are shown as the mean±SEM. p values are indicated.

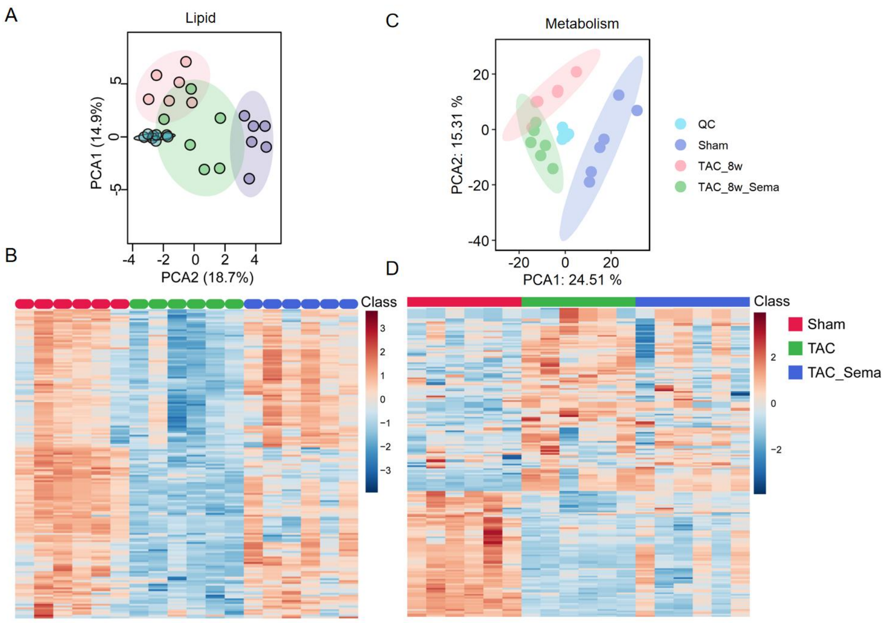


**Figure S4. Effects of 8 weeks of Sema treatment on lipid and energy metabolism in pathological cardiac remodeling.** (A-B) Untargeted metabolomics analysis of lipidomics and metabolomics-related total heatmap and PCA map (n=6). (C-G) Metabolome-based analysis of the energy metabolism-related total heatmap and PCA map (n=6). All results are shown as the mean±SEM. p values are indicated. PCA,principal component analysis.

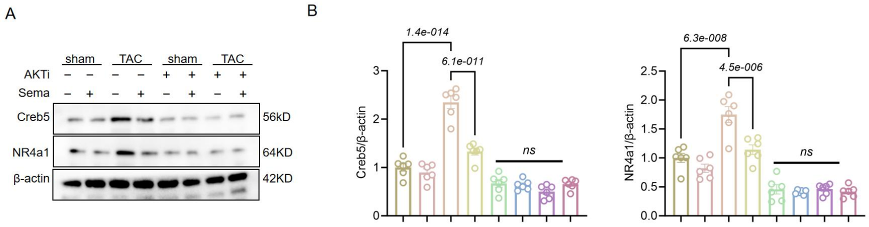


**Figure S5. The therapeutic effects of Sema and elevated Creb5/NR4a1 due to myocardial hypertrophy can be counteracted after the use of AKT inhibitors.** (A) Protein blot levels of Creb5 and NR4a1 under stimulation with AKT inhibitors in the Sema or vehicle groups (n=6 independent experiments with similar results). (B) Quantitative protein map of Creb5 and NR4a1, normalized to β-actin (n=6); Creb5/ β -actin: F (7, 40) = 62.37, P = 1.62e-019; NR4a1/β-actin: F (7, 40) = 39.19, P = 5.96e-016. All results are shown as the mean±SEM, and analysis using one-way ANOVA followed by Bonferroni post hoc test was conducted. p values are indicated. Source data are provided as a Source

Data file.

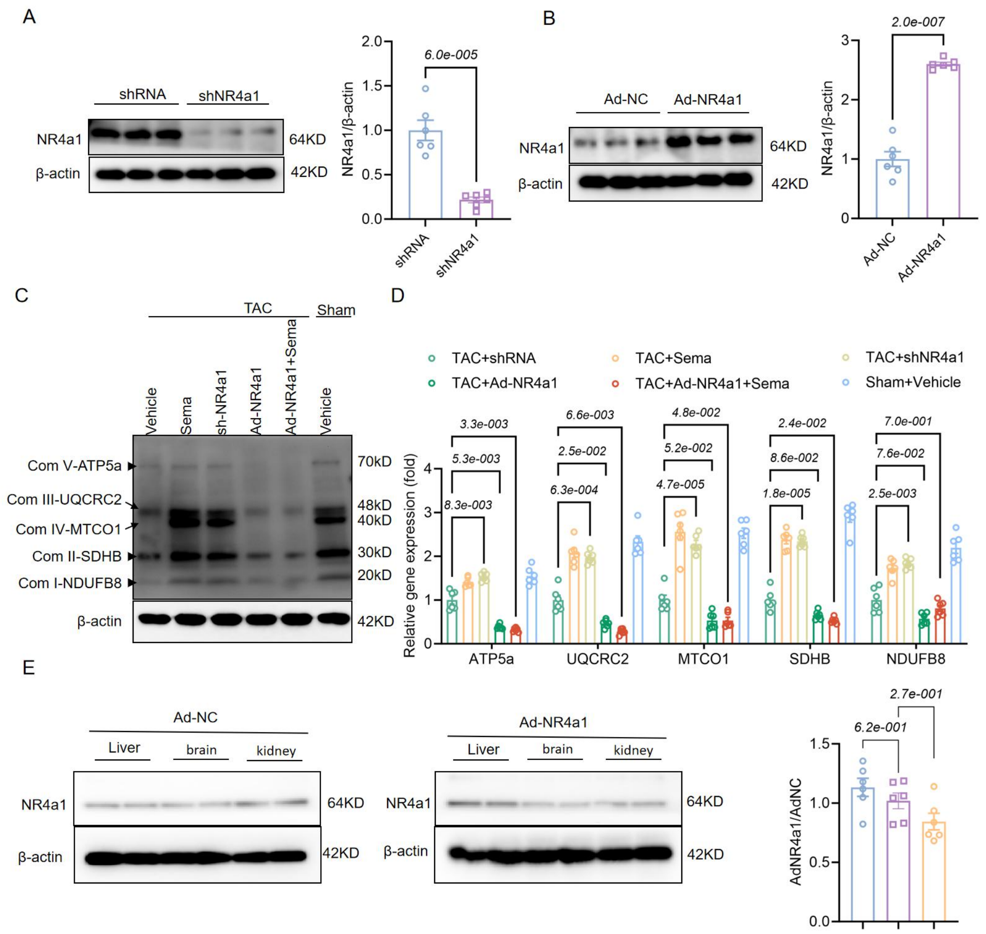


**Figure S6. Effects on mitochondrial function after NR4a1 knockdown or overexpression.** (A) Western blot analysis of NR4a1 knockdown efficiency and its quantification normalized to β-actin (n=6 independent experiments with similar results); t (6) =6.614, p = 6.0e-005. (B) Western blot analysis of NR4a1 overexpression efficiency and its quantification with β-actin as a standard (n=6 independent experiments with similar results); t (6) = 12.49, p=2.0e-007. (C-D) Western blot analysis of NR4a1 overexpression or knockdown of mitochondrial respiratory chain proteins in mice, normalized to β-actin (n=6 independent experiments with similar results); F (20, 120) = 10.53, P = 4.1e-018, F (2.387, 71.62) = 59.11, P = 2.4e-017, F (5, 30) = 207.6, P = 2.5e-022, F (30, 120) = 2.802, P = 4.0e-005. (E) Western blot of protein expression in mouse liver, kidney, brain after NR4a1 overexpression (n=6 independent experiments with similar results); F (2, 15) = 4.237, P = 3.5e-002. All results are shown as the mean±SEM, and analyzed (A, B) using an unpaired two-tailed Student's t test. For the analysis in (D), repeated measures two-way ANOVA followed by Sidak post hoc test was conducted, p values are indicated. Source data are provided as a Source Data file.

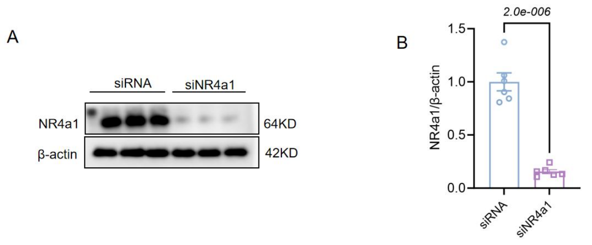


**Figure S7. NR4a1 in vitro knockdown low-efficiency validation.** (A-B). Western blotting map of NR4a1 knockdown efficiency and its quantification normalized with β-actin, n=6 independent experiments with similar results; t (6) = 9.727, p = 2.0e-006. All results are shown as the mean±SEM, and analyzed using an unpaired two-tailed Student′s t test, p values are indicated. Source data are provided as a Source Data file.

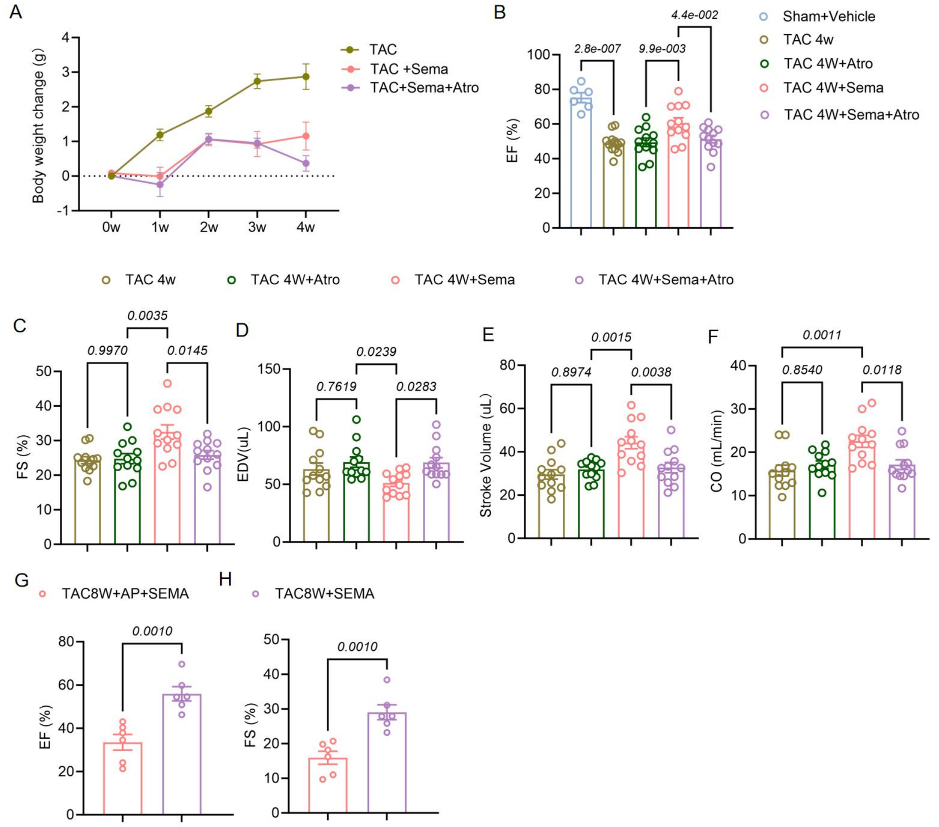


**Figure S8. Cardioprotection induced by Sema is mediated by a muscarinic mechanism.** (A) Effect of Atro on body weight of mice after TAC surgery n=12. (B-E) Cardiac function of mice after TAC 4W (EF, FS, EDV and Stroke Volume) n=12; EF: F (4, 49) = 15.02, P = 4.4e-008; FS: F (3, 44) = 6.564, P = 0.0009; EDV:F (3, 44) = 3.837, P = 0.0159; Stroke Volume: F (3, 44) = 8.680, P = 0.0001. (F) The Cardiac output of mice n=12; F (3, 44) = 6.471, P = 0.0010. (G-H) Cardiac function of mice after TAC 8W (EF and FS) n=6; EF: t (6) =4.565, p = 0.0010; FS: t (6) = 4.599, p =0.0010. All results are shown as the mean ± SEM, and analysis using one-way ANOVA followed by Bonferroni post hoc test (A-F) was conducted. For the analysis in (G and H), using an unpaired two-tailed Student′ s t test. p values are indicated. Source data are provided as a Source Data file. CO, Cardiac output; EF, ejection fractions;

FS, fractional shortening.

**Supplemental Tables**

**Table S1**

| Transcriptomic profiling and metabolism analysis | |
|---|---|
| REAGENT | Uniform Resource Identifier |
| GSEA | http://www.gsea-msigdb.org/gsea |
| Metascope | http://www.metascape.org/gp/index.htmI |
| Cytoscope | http://cytoscape.org |
| R | http://www.r-project.org |
| MetaboAnalyst | http://www.metaboanalyst.ca |
| MetDNA | http://www.metdna.zhulab.cn |
| OSI | http://www.nxhh.net/qkwenda/1460782.html |
| Inter Self-built libraries | / |
| MS-DIAL | http://prime.psc.riken.j.msdial/main.html |

**Western blot images**

**Figure S5A**

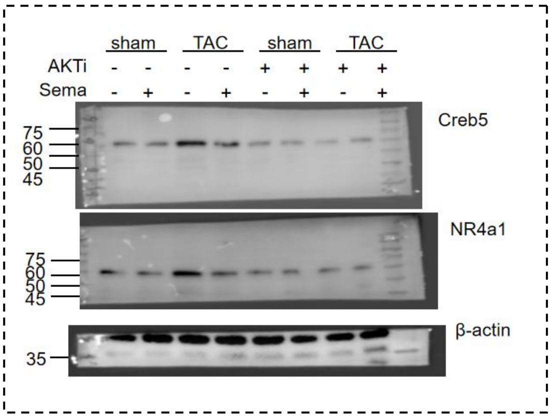

**Figure S6A**

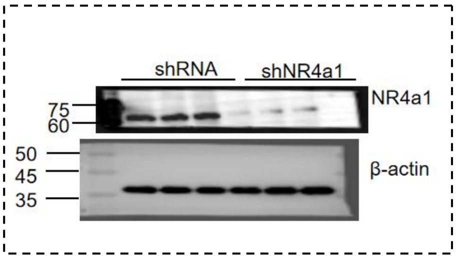


**Figure S6B**

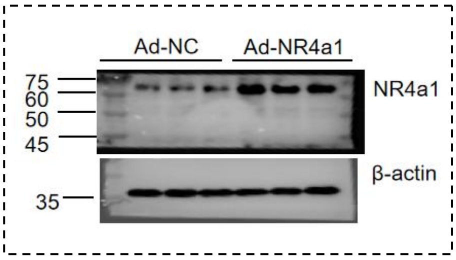


**Figure S6C**

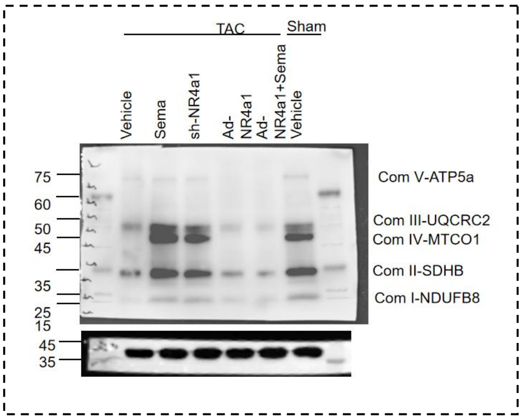


**Figure S6E**

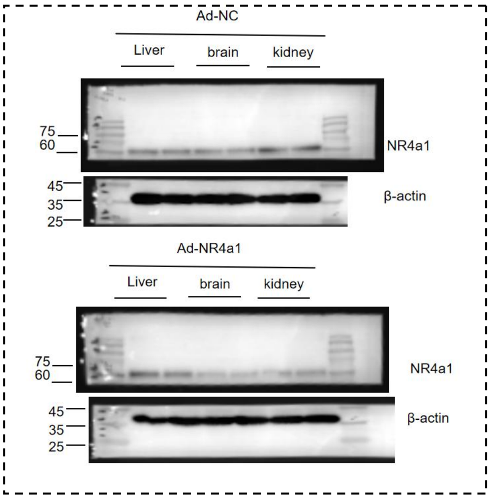


**FigureS7A**

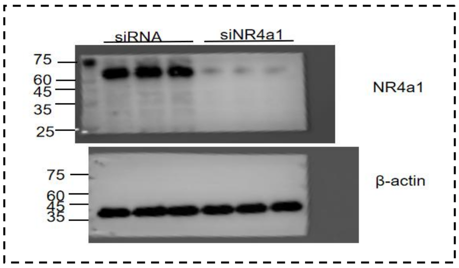

Supplement: Supplementary file 1 — Supplementary Information [file 41467_2024_48970_MOESM1_ESM.pdf]
