# Supplementary material for: Semaglutide ameliorates cardiac remodeling in male mice by optimizing energy substrate utilization through the Creb5/NR4a1 axis
Source: Nat Commun. 2024 Jun 4;15:4757. doi: 10.1038/s41467-024-48970-2 (PMC11150406; doi:10.1038/s41467-024-48970-2)
Supplement: Supplementary file 3 — Description of Additional Supplementary Files [file 41467_2024_48970_MOESM3_ESM.pdf]

Description of Additional Supplementary Files

File Name: Supplementary Data 1

Description:Non-targeted metabolomics-related data

File Name: Supplementary Data 2

Description: Primer Lis

File Name: Supplementary Data 3

Description: GSEA report of our RNA-sequencing data based on hallmark gene sets. GSEA was performed using a two-sided permutation test, using False Discovery Rate(FDR) indicator adjustments were made for multiple comparisons.
