# Supplementary material for: Semaglutide ameliorates cardiac remodeling in male mice by optimizing energy substrate utilization through the Creb5/NR4a1 axis
Source: Nat Commun. 2024 Jun 4;15:4757. doi: 10.1038/s41467-024-48970-2 (PMC11150406; doi:10.1038/s41467-024-48970-2)
Supplement: Supplementary file 7 — Reporting Summary [file 41467_2024_48970_MOESM7_ESM.pdf]

nature portfolio

Corresponding author(s): Qi-Zhu Tang

Last updated by author(s): May 6, 2024

Reporting Summary

Nature Portfolio wishes to improve the reproducibility of the work that we publish. This form provides structure for consistency and transparency in reporting. For further information on Nature Portfolio policies, see our Editorial Policies and the Editorial Policy Checklist.

Statistics

For all statistical analyses, confirm that the following items are present in the figure legend, table legend, main text, or Methods section.

n/a	Confirmed	
☐	☒	The exact sample size (*n*) for each experimental group/condition, given as a discrete number and unit of measurement
☐	☒	A statement on whether measurements were taken from distinct samples or whether the same sample was measured repeatedly
☐	☒	The statistical test(s) used AND whether they are one- or two-sided<br>*Only common tests should be described solely by name; describe more complex techniques in the Methods section.*
☐	☒	A description of all covariates tested
☐	☒	A description of any assumptions or corrections, such as tests of normality and adjustment for multiple comparisons
☐	☒	A full description of the statistical parameters including central tendency (e.g. means) or other basic estimates (e.g. regression coefficient) AND variation (e.g. standard deviation) or associated estimates of uncertainty (e.g. confidence intervals)
☐	☒	For null hypothesis testing, the test statistic (e.g. *F*, *t*, *r*) with confidence intervals, effect sizes, degrees of freedom and *P* value noted<br>*Give P values as exact values whenever suitable.*
☒	☐	For Bayesian analysis, information on the choice of priors and Markov chain Monte Carlo settings
☒	☐	For hierarchical and complex designs, identification of the appropriate level for tests and full reporting of outcomes
☐	☒	Estimates of effect sizes (e.g. Cohen's *d*, Pearson's *r*), indicating how they were calculated

Our web collection on statistics for biologists contains articles on many of the points above.

Software and code

Policy information about availability of computer code

Data collection	Vevo®3100 high resolution Preclinical Imaging 496 System (FUJIFILM Visu alSonics,Toronto, Canada), DP74 fluorescence microscope (OLYMPUS, Tokyo,Japan), ChemiDoc™XRS+ System (Bio-Rad Laboratories, Inc.), Roche LightCycler 480 system, BioTek microplate reader (Winooski, Vermont, USA), NanoPhotometer®spectrophotometer (IMPLEN, CA, USA), Qubit®RNA Assay Kit on a Qubit®2.0 Fluorometer (Life Technologies, CA, USA), NEBNext®UltraTM RNA Library Prep Kit llumina® (NEB, USA), The VAHTS mRNA-seq V8 Library Prep Kit (Illumina), ExionLC™ Series UHPLC (USA), SCIEX-ZenoTOF 7600 system (AB Sciex Pte. Ltd)
Data analysis	Image-Pro Plus 6.0 software (Media Cybernetics, Bethesda, MD, USA), lmag ge Lab Software (version6.0, Bio-Rad Laboratories, Inc.), Image J oftware (version6.0, Bio-Rad, Hercules, CA, USA), GraphPad Prism (version 9.0), Excel

For manuscripts utilizing custom algorithms or software that are central to the research but not yet described in published literature, software must be made available to editors and reviewers. We strongly encourage code deposition in a community repository (e.g. GitHub). See the Nature Portfolio guidelines for submitting code & software for further information.

Data

Policy information about availability of data

All manuscripts must include a data availability statement. This statement should provide the following information, where applicable:

- Accession codes, unique identifiers, or web links for publicly available datasets
- A description of any restrictions on data availability
- For clinical datasets or third party data, please ensure that the statement adheres to our policy

The data supporting the findings from this study are available within the manuscript and its supplementary information. Source data are provided with this paper. Any additional raw data are available from the corresponding author upon reasonable request. The datasets generated for the RNA-seq are available through the Gene Expression Omnibus (https://www.ncbi.nlm.nih.gov/geo/query/acc.cgi?acc=GSE262105). The Metabolomic data generated in this study have been deposited in MassIVE under accession code ID: MSV000094408.

Research involving human participants, their data, or biological material

Policy information about studies with human participants or human data. See also policy information about sex, gender (identity/presentation), and sexual orientation and race, ethnicity and racism.

Reporting on sex and gender	The study did not involve human participants or human data.
Reporting on race, ethnicity, or other socially relevant groupings	The study did not involve human participants or human data.
Population characteristics	The study did not involve human participants or human data.
Recruitment	The study did not involve human participants or human data.
Ethics oversight	The study did not involve human participants or human data.

Note that full information on the approval of the study protocol must also be provided in the manuscript.

Field-specific reporting

Please select the one below that is the best fit for your research. If you are not sure, read the appropriate sections before making your selection.

☒ Life sciences ☐ Behavioural & social sciences ☐ Ecological, evolutionary & environmental sciences

For a reference copy of the document with all sections, see nature.com/documents/nr-reporting-summary-flat.pdf

Life sciences study design

All studies must disclose on these points even when the disclosure is negative.

Sample size	All sample sizes in the experiments were determined based on similar studies in the field by us and other research groups (PMID: 36386478,33042267, PMC9691357, PMC9672960). All sample sizes were sufficiently large for quantitative purposes during the analysis, and differences were observed in past experiments of a similar nature.
Data exclusions	No data were excluded from analysis.
Replication	All experiments used at least three independent samples. Details of the replications of each experiment are provided in the legend, and all replication attempts were successful, yielding similar results.
Randomization	All samples were treated completely randomly throughout the study. All mice were randomly assigned to specific treatment groups.
Blinding	During the experiment, the researchers were blinded to all subgroup information such as group assignment, animal treatment, data collection and analysis.

Reporting for specific materials, systems and methods

We require information from authors about some types of materials, experimental systems and methods used in many studies. Here, indicate whether each material, system or method listed is relevant to your study. If you are not sure if a list item applies to your research, read the appropriate section before selecting a response.

Materials & experimental systems

n/a	Involved in the study
	[x] Antibodies
[x]	Eukaryotic cell lines
[x]	Palaeontology and archaeology
	[x] Animals and other organisms
[x]	Clinical data
[x]	Dual use research of concern
[x]	Plants

Methods

n/a	Involved in the study
[x]	ChIP-seq
[x]	Flow cytometry
[x]	MRI-based neuroimaging

Antibodies

Antibodies used

anti-DRP1 (SANTA SC-32898 1:1000), anti-OPA1 (SANTA sc-30573 1:1000), anti-Mfn1 (SANTA sc-50330 1:1000), anti-Mfn2 (SANTA sc-100560 1:1000), anti-Tom20 (SANTA sc-17764 1:1000), anti-COX IV (CST #11967 1:1000), anti-SDHB (ABclonal A23832 1:2000), anti-NDUFV2 (ABclonal A7442 1:2000), anti-ATP5A1 (proteintech 14676-1-AP 1:1000), anti-VDAC (ABCAM ab191440 1:1000), anti-GLUT1 (ABclonal A11208 1:1000), anti-GLUT4 (ABCAM ab654 1:1000), anti-CD36 (proteintech 18836-1-AP 1:2000), anti-β-Actin (CST #4970 1:1000), anti-PI3K (CST #4257 1:1000), anti-Akt (CST #4691 1:1000), anti-p-Akt (Ser473) (CST #4691 1:1000), anti-NR4a1 (CST #3960 1:1000), anti-Creb5 (ABCAM ab168928 1:1000), anti-P-NR4a1 (CST #5095 1:1000), anti-Lamin B1 (proteintech 12987-1-AP 1:1000), anti-MIRO1 (ABCAM ab188029 1:1000), anti-UQCRC2 (ABclonal A4366 1:1000), anti-MTCO1 (ABclonal A17889 1:1000), anti-NDUFB8 (ABclonal A19732 1:1000), anti-a-actinin (Abcam ab108198 1:1000), goat anti-mouse IgG Alexa Fluor 488 secondary antibodies Invitrog en A11001 IF (1:200); goat anti-rabbit lgG Alexa Fluor 568 secondary antibodies Invitrogen A11011 IF (1:200).

Validation

All antibodies in this study were used and validated according to the provided data sheets and references for the specific technique (western blot, immunostaining or COIP) found directly on the manufacturer's website.
1.anti-DRP1: https://www.scbt.com/zh/p/drp1-antibody-h-300#citations
2.anti-OPA1: https://www.scbt.com/zh/p/opa1-antibody-c-15
3.anti-Mfn1: https://www.scbt.com/zh/p/mfn1-antibody-h-65
4.anti-Mfn1: https://www.scbt.com/zh/p/mfn2-antibody-xx-1
5.anti-COXIV: https://www.cellsignal.cn/products/primary-antibodies/cox-iv-4d11-b3-e8-mouse-mab/11967
6.anti-SDHB: https://abclonal.com.cn/catalog/A23832
7.anti-NDUFV2: https://abclonal.com.cn/catalog/A7442
8.anti-VDAC: https://www.abcam.cn/products/primary-antibodies/vdac1porin-antibody-ab191440.html
9.anti-GLUT1: https://abclonal.com.cn/catalog/A11208
10.anti-GLUT4: https://www.abcam.cn/products/primary-antibodies/glucose-transporter-glut4-antibody-ab654.html
11.anti-CD36: https://ptgcn.com/products/CD36-Antibody-18836-1-AP.htm
12.anti-β-Actin: https://www.cellsignal.cn/products/primary-antibodies/b-actin-13e5-rabbit-mab/4970
13.anti-PI3K: https://www.cellsignal.cn/products/primary-antibodies/pi3-kinase-p85-19h8-rabbit-mab/4257
14.anti-Akt: https://www.cellsignal.cn/products/primary-antibodies/akt-pan-c67e7-rabbit-mab/4691
15.anti-p-Akt: https://www.cellsignal.cn/products/primary-antibodies/phospho-akt-ser473-d9e-xp-174-rabbit-mab/4060
16.anti-NR4a1: https://www.cellsignal.cn/products/primary-antibodies/nur77-d63c5-xp-174-rabbit-mab/3960
17.anti-Creb5: https://www.abcam.cn/products/primary-antibodies/creb5-antibody-ab168928.html
18.anti-p-NR4a1: https://www.cellsignal.cn/products/primary-antibodies/phospho-nur77-ser351-d22g5-rabbit-mab/5095
19.anti-Lamin B1: https://ptgcn.com/products/LMNB1-Antibody-12987-1-AP.htm
20.anti-MIRO1: https://www.abcam.cn/products/primary-antibodies/miro1-antibody-cl1083-ab188029.html
21.anti-UQCRC2:https://abclonal.com.cn/catalog/A4366
22.anti-NDUFB8:https://abclonal.com.cn/catalog/A19732
23.anti-a-actinin:https://www.abcam.cn/products/primary-antibodies/alpha-actinin-4-antibody-epr25332-ab108198.html
24.goat anti-mouse lgG Alexa Fluor 488 secondary antibodies: https://www.thermofisher.cn/cn/zh/antibody/product/Goat-anti-Mouse-lgG-H-L-Cross-Adsorbed-Secondary-Antibody-Polyclonal/A-11001
25.goat anti-rabbit lgG Alexa Fluor 568 secondary antibodies: https://www.thermofisher.cn/cn/zh/antibody/product/Goat-anti-Rabbit-lgG-H-L-Cross-Adsorbed-Secondary-Antibody-Polyclonal/A-11011

Animals and other research organisms

Policy information about studies involving animals; ARRIVE guidelines recommended for reporting animal research, and Sex and Gender in Research

Laboratory animals

All animals were housed in specific pathogen-free (SPF) facilities (20-25°C and 45-55% humidity) and approved by the Animal Welfare Ethics Committee at Renmin Hospital of Wuhan University (No. WDRM20220803B). C57BL/6J male mice were purchased from the Institute of Laboratory Animal Science, Chinese Academy of Medical Sciences (Beijing, China). The 8-week-old male mice were kept in Individually Ventilated Cages with a density of 4-6 mice per cage under a specific pathogen-free. All mice were fed with an irradiated chow diet (#1035 for reproductive feeding and #1025 for maintenance feeding, Beijing HFK Bioscience Co., Ltd, Beijing, China), with free access to drinking water. All mice were sacrificed at indicated times by cervical dislocation.

Wild animals

No wild animals was used in this study.

Reporting on sex	All animal experiments were performed using male mice to ensure that sex difference influences were excluded.
Field-collected samples	No field-collected samples were used in the study.
Ethics oversight	All the experimental procedures were approved by the Animal Care and Use e Committee of Renmin Hospital of Wuhan University, and were also in accordance with the Guidelines for the Care and Use of Labora tory Animals published by the US National Institutes of Health.

Note that full information on the approval of the study protocol must also be provided in the manuscript.

Plants

Seed stocks	*Report on the source of all seed stocks or other plant material used. If applicable, state the seed stock centre and catalogue number. If plant specimens were collected from the field, describe the collection location, date and sampling procedures.*
Novel plant genotypes	*Describe the methods by which all novel plant genotypes were produced. This includes those generated by transgenic approaches, gene editing, chemical/radiation-based mutagenesis and hybridization. For transgenic lines, describe the transformation method, the number of independent lines analyzed and the generation upon which experiments were performed. For gene-edited lines, describe the editor used, the endogenous sequence targeted for editing, the targeting guide RNA sequence (if applicable) and how the editor was applied.*
Authentication	*Describe any authentication procedures for each seed stock used or novel genotype generated. Describe any experiments used to assess the effect of a mutation and, where applicable, how potential secondary effects (e.g. second site T-DNA insertions, mosiacism, off-target gene editing) were examined.*
